# Experiences of Diagnostic Delay and Fertility Anxiety Among Women with Endometriosis: A Qualitative Study

**DOI:** 10.3390/jcm15218510

**Published:** 2026-11-02

**Authors:** Selinay Aktaş Demir, Rüveyda Ölmez Yalazı

**Affiliations:** 1 Department of Midwifery, Faculty of Health Sciences, Istanbul Atlas University, Istanbul 34408, Türkiye; 2 Department of Midwifery, Faculty of Health Sciences, Fenerbahçe University, Istanbul 34758, Türkiye; ruveyda.olmezz@gmail.com

**Keywords:** endometriosis, diagnostic delay, fertility anxiety, qualitative research, reproductive health, women’s health, patient experiences

## Abstract

**Background/Objectives:** Endometriosis often follows a prolonged diagnostic pathway and raises uncertainty about fertility. This study explored how women experienced diagnostic delay, fertility anxiety, healthcare encounters, and coping. **Methods:** In a qualitative descriptive study, 18 women were recruited from the gynecology outpatient clinic of a private hospital in Istanbul, Türkiye. All reported a previous physician diagnosis of endometriosis and an interval of at least two years between symptom onset and diagnosis. Individual semi-structured interviews were audio-recorded, transcribed, and analyzed with a collaboratively developed thematic codebook. **Results:** Participants’ mean age was 30.3 ± 4.8 years, and their mean self-reported diagnostic interval was 7.9 ± 3.6 years. Five themes addressed experiences of diagnostic delay; the meaning and consequences of diagnosis; fertility anxiety and reproductive decisions; healthcare experiences and expectations; and social support and coping. Women described the normalization of severe pain, dismissive or fragmented care, and the need to persist in seeking answers. Diagnosis validated symptoms but also brought the perceived costs of lost time into focus. Some participants described concerns about future fertility before attempting conception, while others discussed anxiety during pregnancy attempts; these concerns shaped treatment choices, relationships, and future plans. Participants described anxiety about ovarian reserve, unsuccessful fertility treatment, and pressure around marriage or motherhood; they sought clearer explanations and continuity of care. **Conclusions:** In this sample, diagnostic delay and fertility anxiety were intertwined with social expectations and healthcare experiences. Timely recognition of disabling symptoms, individualized fertility information, validating communication, and coordinated care may address needs across the endometriosis pathway.

## 1. Introduction

Endometriosis is a chronic, estrogen-dependent inflammatory condition characterized by endometrium-like tissue outside the uterine cavity. It affects an estimated 10% of women of reproductive age and is associated with dysmenorrhea, chronic pelvic pain, dyspareunia, fatigue, bowel or urinary symptoms, and infertility [1,2]. The clinical presentation is heterogeneous, symptom severity does not consistently correspond to the anatomical extent of disease, and no single symptom is specific to endometriosis. These characteristics complicate recognition and contribute to substantial variation in diagnostic pathways. Current European Society of Human Reproduction and Embryology guidance therefore recommends that endometriosis be considered in individuals presenting with cyclical or non-cyclical pelvic pain, deep dyspareunia, dyschezia, dysuria, fatigue, or infertility and emphasizes individualized diagnostic and treatment decisions [3].

Delayed diagnosis remains common in endometriosis. Recent reviews indicate that the interval between symptom onset and diagnosis varies considerably across countries and study populations and may extend for many years [4,5,6]. Diagnostic delay develops through interactions among patient-level, interpersonal, clinical, and health-system factors. Menstrual pain may be normalized by women, families, schools, and communities; symptoms may be concealed because menstruation and reproductive health remain stigmatized; and affected women may initially rely on self-management rather than seeking care [4,6]. Once healthcare is accessed, nonspecific symptoms, overlap with gastrointestinal or urinary disorders, limited professional awareness, repeated empirical treatment, delayed specialist referral, and the absence of a universally reliable non-invasive test may further prolong the process [3,4,5,6].

Beyond physical symptoms, endometriosis can affect multiple areas of life. International research has documented marked reductions in health-related quality of life, participation in education and employment, work productivity, social functioning, intimate relationships, and emotional well-being [7,8,9,10,11]. Women may miss school or work, restrict daily activities, alter career plans, and withdraw from social or sexual relationships because of pain and uncertainty [8,9,10]. Compared with healthy controls, women with endometriosis report more symptoms of depression and anxiety; these symptoms are associated with chronic pain, fatigue, poor sleep, and impaired sexual functioning [11]. Thus, endometriosis is increasingly understood not only as a gynecological disorder but also as a long-term biopsychosocial condition requiring coordinated and person-centered care.

Fertility is a particularly important dimension of this burden. Endometriosis may be identified during an infertility evaluation, and women may face uncertainty about fertility and reproductive options [3,12]. Fertility concerns may arise alongside decisions about hormonal treatment, surgery, fertility preservation, and reproductive planning [3,12]. For women undergoing assisted reproductive treatment, repeated procedures and unsuccessful attempts may intensify grief, uncertainty, and perceived loss of control [12,13,14]. Fertility-related distress therefore cannot be reduced to a clinical diagnosis of infertility; it also includes anticipated loss, reproductive time pressure, altered life trajectories, strain within couples, and uncertainty about future choices [12,13].

Interactions with healthcare professionals can either intensify or alleviate these difficulties. Qualitative studies have repeatedly described experiences of symptoms being trivialized, psychologized, or attributed to ordinary menstruation [15,16,17,18]. Such encounters may undermine trust and require women to assume responsibility for researching the condition, requesting referral, and repeatedly advocating for further investigation [15,18]. Conversely, being listened to, receiving a coherent explanation, and participating in decisions can validate women’s experiences and restore confidence in care. Existing guidance and qualitative evidence support clear information, continuity, psychological support, and coordinated multidisciplinary care [3,15,19].

Although the international literature increasingly documents diagnostic delay and the broad impact of endometriosis, experiences are shaped by sociocultural expectations, health-system organization, access to specialist services, and norms surrounding menstruation, marriage, sexuality, and motherhood [4,6,17]. In Türkiye, studies have documented quality-of-life and sexual-health effects and validated a Turkish endometriosis-specific quality-of-life instrument [20,21]. Özcan et al. [22] examined anxieties and coping strategies among women with endometriosis in Türkiye, including concerns about pregnancy, ovarian damage, delayed diagnosis, and limited information. The present study focuses on women reporting a diagnostic interval of at least two years and examines how that pathway intersects with fertility concerns, healthcare encounters, intimate and family relationships, and coping. Exploring these experiences in their sociocultural context is essential for developing responsive clinical communication and support strategies.

Accordingly, this study aimed to explore the experiences of women with endometriosis regarding diagnostic delay and fertility anxiety. It also examined the perceived consequences of delayed diagnosis, experiences and expectations regarding healthcare, and the social and personal strategies used to cope with the condition. By centering women’s narratives, the study sought to identify modifiable barriers and support needs relevant to person-centered and multidisciplinary endometriosis care.

## 2. Materials and Methods

### 2.1. Study Design

We used a qualitative descriptive design to examine women’s accounts of the time before an endometriosis diagnosis and their anxiety about fertility [23]. Individual semi-structured interviews allowed participants to describe their symptoms, healthcare encounters, diagnosis, and reproductive decisions in their own words. The transcripts were examined using a team-based codebook approach to thematic analysis [24]. Reporting was guided by the 32-item Consolidated Criteria for Reporting Qualitative Research (COREQ) checklist [25]; the COREQ 32-Item Checklist is provided in Supplementary Table S1.

### 2.2. Participants and Setting

Participants were recruited from the gynecology outpatient clinic of a private hospital in Istanbul, Türkiye, between 22 June and 21 August 2026. Women were eligible to participate if they were 18–40 years of age, self-reported that a physician had diagnosed them with endometriosis at least six months before the interview, reported a diagnostic delay of at least two years between symptom onset and diagnosis, were able to communicate in Turkish, and provided written consent to participate in an audio-recorded interview. Eligibility was based on the participant’s account; medical records, imaging findings, and surgical or histopathological confirmation were not independently reviewed by the research team.

Eligibility screening was conducted before the interviews and separately from the descriptive form and interview guide in Supplementary Table S2. The researchers asked whether a healthcare professional had attributed any fertility difficulty exclusively to a cause unrelated to endometriosis, such as an isolated male factor. Women who reported such an attribution met the infertility-related exclusion criterion. They were also excluded if they had a severe psychiatric or cognitive condition that could prevent meaningful participation in the interview. The infertility-related criterion was used to keep the accounts focused on fertility anxiety that participants associated with endometriosis. These reports, including any partner-related diagnosis, were not independently verified by the research team.

Women attending the gynecology outpatient clinic were asked by clinic staff whether they had previously been diagnosed with endometriosis and whether they were willing to receive information about the study. Women who answered affirmatively and expressed interest were referred to the two researchers (S.A.D. and R.O.Y.), who assessed the remaining eligibility criteria, explained the purpose and procedures, and invited eligible volunteers to participate. Eighteen women who volunteered and met the eligibility criteria provided written informed consent and completed an interview; no enrolled participant withdrew. The numbers of women approached, those who declined before consent, and those excluded during eligibility screening were not recorded. The researchers were not involved in participants’ clinical care and had no prior relationship with them.

Purposive, criterion-based sampling was used to recruit women who met the eligibility criteria and could provide direct, information-rich accounts relevant to the study aim [26]. The strategy did not use prespecified quotas or formal maximum-variation sampling. Nevertheless, the resulting sample encompassed variation in age, marital status, educational attainment, employment status, time from symptom onset to diagnosis, surgical history, fertility history, and treatment experience (Table 1), allowing comparison across different diagnostic and reproductive trajectories within the defined population.

Recruitment took place alongside preliminary analysis. Following each interview, S.A.D. and R.O.Y. examined the emerging codes in relation to those from earlier interviews and considered whether the new account added a substantive code, a further dimension to an existing code, or a meaningful connection between developing patterns [27]. The final interviews reinforced patterns already documented and did not prompt a substantive change to the codebook or developing themes. Both researchers agreed that further interviews were unlikely to extend the analysis in ways relevant to the study aim, and recruitment ended after 18 interviews. Interview-by-interview comparisons and the decision to stop were documented in the evolving codebook and analytic notes.

### 2.3. Data Collection

S.A.D. and R.O.Y., both female academic nurses experienced in women’s health practice and qualitative research, conducted the interviews. Neither had a pre-existing relationship with participants or a role in their clinical care. Before each interview, participants were told that the researchers worked independently of their treatment team and that their decision to participate would not affect their care. Participants were also told that they could pause or end the interview if they became distressed and that the researchers could direct them to clinical support if needed. No participant required a pause, early termination, or referral for distress. Each woman took part in one face-to-face interview in a private room at the gynecology outpatient clinic, where interruptions were minimized and no one else was present. The interviews lasted approximately 50–60 min and were audio-recorded with permission. During and immediately after each interview, the interviewer wrote brief field notes about the setting and initial analytic impressions; the researchers later considered these notes alongside the transcripts.

The semi-structured interview guide was developed by the research team using the literature on the clinical diagnosis of endometriosis [28], women’s psychological experiences [29], and the effects of endometriosis on quality of life and sexual life [21]. It comprised a 12-item descriptive information form and five main open-ended questions (Supplementary Table S2). The descriptive form included items on age, educational level, marital and employment status, the self-reported interval from first symptoms to diagnosis, childbearing status, endometriosis-related surgery, previous attempts to conceive, information received about possible reduction in ovarian reserve, fertility treatment history, time since diagnosis, and current treatment or follow-up.

The five main open-ended questions invited participants to describe their path from first symptoms to diagnosis, what they experienced during that period, how endometriosis affected their thoughts, feelings, or plans regarding fertility or having children, whether they had experienced fertility anxiety or uncertainty, how they dealt with those feelings, and what approach or support they wanted from healthcare professionals or services. Main questions were supplemented with prompts tailored to each participant’s responses. At the end of the interview, participants were asked whether they wished to add anything about their endometriosis experience, diagnostic pathway, or fertility anxiety.

Before the main interviews, four women with endometriosis took part in a pilot test of the interview guide. Their feedback helped the team assess whether the questions were clear, understandable, and appropriately ordered; the guide was then finalized. None of the four pilot participants was included in the main study sample.

The interview recordings were transcribed word for word in Turkish, the language in which the interviews took place. The transcripts were not sent to participants for review or correction. Once the analysis was complete, S.A.D. and R.O.Y. each prepared an English translation of the final themes, subthemes, and selected participant quotations. They compared their versions item by item and discussed differences until they agreed on wording that conveyed the meaning and sociocultural context of the original Turkish accounts.

### 2.4. Data Analysis

Data were analyzed using a codebook approach to thematic analysis, organized using the six analytic phases originally described by Braun and Clarke [30]. The six phases served as a procedural guide; the shared codebook, independent coding, and consensus discussions reflect a collaborative codebook approach rather than reflexive thematic analysis. The selection and description of a codebook approach, including its distinction from reflexive thematic analysis, were guided by Braun and Clarke’s [24] comparison of pattern-based qualitative analytic approaches. The shared coding framework supported team-based analysis [31]. Coding was performed manually without qualitative data-analysis software.

S.A.D. and R.O.Y. each read the transcripts repeatedly and independently assigned initial codes. They compared these codes and, through discussion, established a codebook for use across the dataset. When their coding differed, they discussed the relevant passages and reached a shared decision. The researchers then brought related codes together into provisional themes, examined and refined those themes, and agreed on their definitions and names.

The interview guide defined broad areas of inquiry rather than a predetermined set of codes or themes. Initial codes were concise, data-near labels assigned to passages relevant to the study aim in the Turkish transcripts. The two researchers compared their coded passages, grouped codes with related meanings into intermediate categories, refined these into subthemes, and integrated related subthemes into the five final themes. Supplementary Table S3 traces the progression from initial codes to intermediate categories, subthemes, and final themes. The five final themes retained a descriptive connection to the interview topics, while patterns linking these areas were considered across accounts and synthesized in the Discussion.

### 2.5. Trustworthiness

We considered credibility, dependability, confirmability, and transferability using the criteria described by Guba [32] and Korstjens and Moser [33]. Credibility was supported by independent coding, discussion to reach consensus, field notes, direct participant quotations, and review of the final themes by two external experts. The evolving codebook and analytic notes documented recruitment, pilot testing, data collection, transcription, comparisons between interviews, coding decisions, and theme development to provide an audit trail for dependability. Two weeks after the initial coding of the full dataset, both researchers coded it again and compared the results with the shared codebook and developing themes. Reflexive discussions and the external review supported confirmability. The external reviewers were an academic nurse experienced in qualitative methodology and an academic nurse specializing in women’s health and gynecological nursing. Their role was limited to reviewing the coding tree, final themes, and selected supporting quotations; they did not code transcripts, develop the codebook, or make final analytic decisions. We described the setting, participants, eligibility criteria, sampling, interviews, and analysis in detail to help readers assess transferability. Neither transcripts nor findings were returned to participants, so participant checking was not undertaken.

### 2.6. Researcher Characteristics and Reflexivity

The research team comprised two female academic nurses specializing in women’s health and diseases. S.A.D. holds an MSc degree, and R.O.Y. holds a PhD degree. Both had clinical and qualitative research experience in women’s health and held academic appointments in departments of midwifery at the time of the study. Both researchers conducted interviews, independently coded the transcripts, compared codes and themes, and contributed to the methods and findings. During data collection and analysis, S.A.D. and R.O.Y. used reflexive discussions to examine how their professional perspectives might influence interpretation [34]. They evaluated analytic decisions together. As women’s health professionals, the researchers recognized that their clinical backgrounds could shape attention to healthcare dismissal, fertility anxiety, and support needs. These assumptions were discussed reflexively during analysis.

### 2.7. Ethical Considerations

The Fenerbahçe University Non-Interventional Clinical Research Ethics Committee approved the study on 9 June 2026 (protocol no. 73.2026fbu), and the private hospital granted institutional permission. The research followed the Declaration of Helsinki. Before being interviewed, each woman was informed about the study’s purpose and procedures, confidentiality, the voluntary nature of participation, and her right to withdraw at any time without giving a reason. All participants provided written informed consent, including consent to audio recording and publication of anonymized quotations. Personal identifiers were replaced with participant codes in the transcripts and research reports.

## 3. Results

The study included 18 women with a mean age of 30.3 ± 4.8 years (range: 22–39 years). The mean interval from symptom onset to the diagnosis reported by the participant was 7.9 ± 3.6 years (range: 2–15 years). Thirteen participants (72.2%) were married, eleven (61.1%) were employed, and four (22.2%) had children. Twelve women (66.7%) had attempted pregnancy, six (33.3%) had received fertility treatment, twelve (66.7%) had received information about ovarian reserve, and two (11.1%) had undergone surgery for endometriosis. Further sociodemographic and clinical characteristics are presented in Table 1.

The interviews yielded five themes: (1) experiences of diagnostic delay; (2) meaning and consequences of the delayed diagnosis; (3) fertility anxiety and reproductive decision-making; (4) experiences and expectations regarding healthcare; and (5) social support and coping strategies. Table 2 lists the subthemes within each theme. The sections that follow describe these patterns using participants’ accounts and illustrative quotations; Supplementary Table S3 shows how the codes were organized into the final thematic structure. The quotations below illustrate the analytic points; their number and distribution do not indicate how many participants contributed to each subtheme.

Across the five themes, reproductive intention and position in the diagnostic pathway provided important experiential contexts. The accounts presented below illustrate repeated monitoring and treatment burden among women attempting conception, and prospective uncertainty and perceived time pressure among women who had not attempted conception. Pain, social expectations, information gaps, and the need for coordinated support crossed these contexts.

### 3.1. Experiences of Diagnostic Delay

#### 3.1.1. Normalization and Delayed Help-Seeking

P18 and P16 described family explanations that normalized severe menstrual pain or restricted care seeking while unmarried. In P18’s account, this delayed a first consultation; P16 described a family concern about pelvic examination. These cases illustrate how family beliefs could postpone help-seeking. One participant explained:

“My mother said, ‘We had it too; it will pass when you get married.’ That is why I did not go to the doctor for two years.”

(P18)

Another participant described how family beliefs restricted her access to care until marriage:

“My mother said, ‘A girl does not go to the doctor or get examined.’ She was very afraid that something would happen to my hymen. I could only go after I got married.”

(P16)

#### 3.1.2. Clinical Dismissal and Symptom Misattribution

The accounts below illustrate two forms of missed recognition: repeated emergency treatment without diagnostic continuity (P3) and psychological attribution of pain (P8). One participant stated:

“I went to the emergency department countless times; every time they gave me an injection and sent me home.”

(P3)

The effects of psychological attribution were particularly enduring for one participant:

“For years they said it was psychological and gave me antidepressants, but I was not depressed; I was in pain. Then, of course, I really became depressed because of the pain.”

(P8)

#### 3.1.3. Patient-Led Recognition and Persistence

P7 and P10 described learning about endometriosis through their own searches and raising the possibility with clinicians. Their accounts illustrate how patients could initiate the diagnostic discussion. A young participant explained:

“I feel as if I diagnosed myself. If I had not said, ‘Could this be endometriosis?’ it would have taken even longer.”

(P7)

Another participant similarly described initiating the diagnostic discussion after researching her symptoms:

“I asked the emergency doctor, ‘Could I have endometriosis?’ I had read about it on the internet; then the diagnosis was made with magnetic resonance imaging.”

(P10)

#### 3.1.4. Fertility Difficulty as a Route to Diagnosis

P2 described diagnosis during infertility evaluation rather than in response to pain, while P13 questioned why endometriosis had not been identified before insemination. These accounts suggest that fertility investigation could make previously overlooked symptoms clinically consequential. One participant explained:

“I was diagnosed through infertility, not through my pain. That felt very strange to me.”

(P2)

Another participant questioned why the condition had not been investigated before fertility treatment:

“Why they did not see this before the insemination, I still do not understand.”

(P13)

Taken together, the four subthemes indicate that diagnostic delay developed through successive rather than isolated barriers. Familial normalization and restrictions related to unmarried status delayed initial help-seeking, whereas dismissal, misattribution, and fragmented referrals prolonged the process after women entered care. Patient self-advocacy and fertility investigations eventually facilitated recognition, but both required women to carry much of the responsibility for moving the diagnostic process forward.

### 3.2. Meaning and Consequences of the Delayed Diagnosis

#### 3.2.1. Relief and Validation After Diagnosis

P1 and P9 described diagnosis as validating pain that had previously been doubted or minimized. Naming the condition also allowed P9 to explain her illness to others. One participant stated:

“When it had a name, I said, ‘So I was not making it up.’”

(P1)

A participant who had struggled to make her pain credible similarly explained:

“Now I can put a name to my pain. I can tell my neighbors and my husband, ‘I have an illness.’ Before, no one believed me.”

(P9)

#### 3.2.2. Anger, Regret, and Lost Time

Relief could coexist with anger about delayed recognition and lost time. P1 described this ambivalence, while P16 reflected on family normalization of symptoms. One participant described the diagnosis as having two opposing meanings:

“For me, the diagnosis means both liberation and lost years.”

(P1)

Self-blame sometimes extended to family members who had normalized symptoms, although women also recognized that their relatives lacked information:

“Sometimes I blame my mother, and then I think I should not blame her; she did not know either.”

(P16)

#### 3.2.3. Disruption of Education, Work, Family, and Intimate Life

The delayed diagnosis was embedded in substantial functional loss. Participants reported missing school or examinations, extending their education, leaving postgraduate study, losing promotion opportunities, reducing productivity, leaving employment, and concealing symptoms because of fear of dismissal. Pain also restricted housework, parenting, social participation, and sexual intimacy. One participant explained how repeated cancellations changed her social identity:

“I cannot go out with my friends; I am always the person who cancels.”

(P3)

Another participant linked pain and treatment demands to occupational and financial consequences:

“I had to leave my job because the treatment process and work could not continue together. When I stopped working, we also struggled financially.”

(P13)

#### 3.2.4. Ongoing Uncertainty and Mistrust

Diagnosis did not resolve all uncertainty. P5 connected delayed diagnosis with mistrust of healthcare, while P14 described uncertainty about whether surgery would be needed. One participant stated:

“Delayed diagnosis means mistrust to me. I can no longer fully trust the healthcare system.”

(P5)

For another participant, uncertainty about deep endometriosis was more burdensome than pain itself:

“No one can say whether the lesion in my bowel will shrink or whether I will need surgery. That uncertainty tires me more than the pain.”

(P14)

The consequences of diagnosis were therefore ambivalent. Naming the disease restored credibility and reduced self-doubt, but it also made the cumulative costs of delay visible. Participants did not describe diagnosis as the end of their illness trajectory; rather, it marked a transition from unexplained pain to chronic uncertainty about recurrence, treatment, function, and future loss.

### 3.3. Fertility Anxiety and Reproductive Decision-Making

#### 3.3.1. Anticipatory Fear and Perceived Reproductive Time Pressure

Three participants who had not attempted conception (P1, P7, and P12) described anticipatory concerns about fertility, while other accounts concerned ongoing attempts to become pregnant. Information about diminished ovarian reserve, disease progression, or possible surgery created a sense of limited reproductive time. Younger participants described being required to consider egg freezing, future disclosure, and motherhood earlier than expected. One unmarried participant stated:

“When I learned that my ovarian reserve was low for my age, I panicked. It was as if a clock had started ticking.”

(P1)

A 22-year-old participant similarly described the premature emotional burden of fertility risk:

“I do not even have a partner, but I am afraid that I may not have a child. Experiencing this at 22 is very strange.”

(P7)

#### 3.3.2. Repeated Disappointment During Attempts to Conceive

For P2, menstruation became a recurring moment of grief; P11 described repeated hope and disappointment during fertility treatment. Their accounts illustrate the emotional burden of unsuccessful attempts. One participant explained:

“Every time I get my period, I go through a mourning process.”

(P2)

Another participant described the cumulative effect of fertility treatment:

“With every attempt I become hopeful and then collapse. Sometimes I try to prepare myself for the possibility that it may never happen, but accepting that is very difficult.”

(P11)

#### 3.3.3. Motherhood Expectations and Feelings of Inadequacy

P16 described an in-law’s comment that made her feel deficient, while P15 described guilt about not giving her child a sibling. These accounts show how reproductive uncertainty intersected with expectations of motherhood. One participant stated:

“My mother-in-law says, ‘There are no infertile women in our family.’ I feel very bad, as if I am an incomplete woman.”

(P16)

A participant experiencing secondary infertility described simultaneous gratitude and guilt:

“Sometimes I look at my son and feel grateful, and sometimes I feel guilty that I may not be able to give him a sibling.”

(P15)

Another mother described how having a child had shaped her expectations about future fertility:

“Because I already had a child, it had never occurred to me that I might have difficulty with fertility.”

(P4)

#### 3.3.4. Tension Between Symptom Treatment and Fertility Goals

Participants described competing treatment priorities when hormonal suppression could reduce pain but was incompatible with attempts to conceive. Surgery could address severe symptoms or disease progression while also creating anxiety about ovarian reserve. Advice that pregnancy might improve endometriosis made reproductive timing feel clinically directed rather than personally chosen. One participant summarized this conflict:

“It is either medication or a baby; the two do not happen together. I endure the pain with painkillers.”

(P6)

Another participant explained how endometriosis altered decisions that she and her spouse had expected to make independently:

“We wanted to travel and focus on our careers for a few years. Now we are asking whether we should have a child early. It feels as if the disease is deciding, not us.”

(P12)

The fertility subthemes formed a continuum from anticipated risk to embodied reproductive loss. In the accounts of P1, P7, and P12, concern about possible future fertility arose before any attempt to conceive; women attempting conception described anxiety linked to repeated menstrual cycles, procedures, and treatment outcomes. Across both contexts, social expectations and competing treatment goals narrowed the sense that reproductive timing remained a fully personal choice.

### 3.4. Experiences and Expectations Regarding Healthcare

#### 3.4.1. Uneven Information About Endometriosis and Ovarian Reserve

The descriptive form recorded ovarian reserve information for 12 participants and no such information for six. P16 questioned why she had not been counselled; P13 described information arriving too late to guide her choices. One participant who had not been counselled asked:

“No one told me anything about ovarian reserve. Now that you ask, I wonder: was I supposed to ask?”

(P16)

Another participant described delayed information as a missed opportunity:

“If someone had explained earlier how this disease could affect my ovaries and that time mattered, perhaps I would have acted sooner.”

(P13)

#### 3.4.2. Importance of Clear and Individualized Communication

P9 valued visual and written explanations during a consultation:

“The doctor explained it by drawing on paper. The nurse wrote down one by one how I should use the medications. It was the first time I felt that a doctor gave me time.”

(P9)

Information was not necessarily experienced as harmful when it was clear and connected to a plan. A participant who received realistic age-related counselling stated:

“It was painful, but it was necessary.”

(P15)

#### 3.4.3. Need for Psychological Support

P2 and P14 described psychological needs left outside routine care. P2 recalled no emotional support after an unsuccessful fertility treatment, and P14 said psychological support had not been discussed. One participant stated:

“When the in vitro fertilization attempt failed, they only said, ‘We will look at the next one.’ I went home and cried for hours. I wish a nurse or psychologist had spoken with me.”

(P2)

Another participant described the biomedical focus of her care:

“Psychological support was never discussed, as if only my body was being cared for.”

(P14)

#### 3.4.4. Need for Coordinated Multidisciplinary Care and Nursing Support

Participants described pain care, fertility services, surgical planning, and psychological support as separate pathways. P5 and P17 requested access to a nurse who could provide information and continuity between appointments. One participant explained:

“For pain you go to one place, for fertility to another, and for psychological support somewhere else. No one looks at the whole. I wish there were an endometriosis nurse I could always reach.”

(P5)

Another participant emphasized the need for a professional who could answer questions between brief medical appointments:

“I wish there were a nurse who would sit and explain this disease and whom I could ask my questions.”

(P17)

Healthcare experiences ranged from dismissive and fragmented encounters to communication that restored confidence and agency. Lack of information and psychological attention contributed to mistrust, while individualized explanation, nurse accessibility, and multidisciplinary assessment represented a preferred alternative. Participants’ expectations centered on continuity and navigation as much as the amount of information provided.

### 3.5. Social Support and Coping Strategies

#### 3.5.1. Support and Strain Within Partner and Family Relationships

Supportive partners attended appointments, administered fertility injections, defended women against relatives, and assumed household tasks during painful periods. Some partners became more understanding after the diagnosis was explained by a clinician. However, prolonged pain or difficulty conceiving also produced fatigue, disagreement, and mismatched coping within couples. One participant stated:

“The doctor called my husband in and explained it to him; then he changed. Now he cooks on the days when I am in pain.”

(P9)

A participant seeking a second child described feeling alone despite her spouse’s accepting position:

“My husband says, ‘We have one child; that is enough.’ It should comfort me, but sometimes it makes me feel as if I am the only one who wants another child.”

(P15)

#### 3.5.2. Information-Seeking, Peer Support, and Self-Management

Participants used online communities, patient groups, symptom-tracking applications, exercise, dietary modification, meditation, art, and psychotherapy to manage pain and uncertainty. Peer groups helped women recognize shared experiences and reduced isolation, while symptom calendars and lifestyle changes created a sense of practical control. One participant stated:

“I read articles and follow specialists. Knowledge gives me a sense of control.”

(P5)

Another participant emphasized the relational value of online groups:

“I follow endometriosis groups on social media. Seeing that I am not alone feels good.”

(P17)

#### 3.5.3. Concealment, Withdrawal, and Protection from Social Pressure

Some women concealed fertility treatment at work, delayed disclosure to partners or families, or stopped discussing pain after repeated minimization. Others withdrew from social visits or friends with children to avoid intrusive questions and painful comparison. Concealment reduced immediate exposure to judgment but also limited access to support. One participant stated:

“My friends say, ‘Everyone has pain,’ so I stopped telling them.”

(P3)

Another participant described withholding fertility information from both families:

“We have not told our families about fertility yet; we are afraid of their pressure.”

(P12)

#### 3.5.4. Use of Complementary and Spiritual Practices

The accounts below illustrate a family-recommended remedy that was not disclosed to a clinician (P6) and the use of a remedy despite uncertainty about its effectiveness (P16). One participant explained:

“My mother-in-law made me drink onion water for a month. I did not tell the doctor because I thought the doctor would be angry.”

(P6)

Another participant described uncertainty rather than confidence in the remedy:

“I do not know whether it works, but I use it thinking, ‘What if it helps?’”

(P16)

Coping was neither uniformly protective nor solely individual. Supportive relationships, peer connection, knowledge, and structured self-management could strengthen agency, while social pressure encouraged concealment and withdrawal. Complementary and spiritual practices offered action in the face of uncertainty but sometimes occurred without clinical discussion, indicating the importance of nonjudgmental enquiry about all treatments and products used.

## 4. Discussion

This qualitative study provides a detailed account of how diagnostic delay, reproductive uncertainty, healthcare encounters, and social context became interwoven in the accounts of women who reported a diagnostic interval of at least two years. Five themes were identified: experiences of diagnostic delay; the meaning and consequences of delayed diagnosis; fertility anxiety and reproductive decision-making; experiences and expectations regarding healthcare; and social support and coping strategies. Rather than representing separate stages, these themes formed a connected illness trajectory. Menstrual pain was initially normalized within families and communities, symptoms were subsequently minimized or misattributed in healthcare settings, and women often had to persist or independently raise the possibility of endometriosis. Diagnosis offered validation, yet it also made lost time and possible reproductive consequences more visible. These experiences shaped trust in healthcare, intimate and family relationships, treatment decisions, and strategies for regaining control.

The accounts of P18 and P16 illustrate how family messages normalized severe menstrual pain and how concern about examination while unmarried could delay care. These findings closely correspond with the qualitative systematic review by Davenport et al. [4], which identified difficulty distinguishing pathological symptoms from normal menstruation, menstrual stigma, self-management, limited professional awareness, and delayed referral as major barriers. They are also consistent with the social-ecological synthesis of Fryer et al. [6], which suggests that delay emerges through interacting individual, interpersonal, organizational, and structural influences rather than a single decision by the patient or clinician. The accounts of P18 and P16 raise the possibility that education addressing disabling menstrual pain and concerns about seeking care before marriage could be useful in this context; whether such education changes help-seeking requires evaluation.

Participants’ accounts of repeated reassurance, short-term symptom treatment, psychological attribution, and alternative diagnoses also parallel recent evidence. Requadt et al. [16] found that participants commonly described symptom normalization, dismissal, and long intervals both before and after first presenting to a clinician. Van der Zanden et al. [18] similarly identified young age, inadequate appraisal in primary care, lack of professional awareness, and the need for women to persist in requesting specialist assessment. The present findings extend this literature by showing that patient self-advocacy can become an informal diagnostic mechanism: some women learned about endometriosis from friends or online sources and initiated the diagnostic discussion themselves. Although such agency can facilitate recognition, it also transfers a disproportionate burden of diagnostic navigation to women who are already managing pain and uncertainty.

The accounts of P2 and P13 show how fertility assessment, or a missed opportunity before insemination, could become part of the route to diagnosis. Their accounts illustrate the paradox that persistent pain had not prompted sustained investigation, whereas difficulty conceiving made symptoms clinically consequential. This pattern is important because it may communicate that women’s pain becomes legitimate primarily when reproduction is threatened. Current evidence shows that diagnostic intervals remain substantial and highly variable despite improved awareness and guidance [5], and recent patient-reported data demonstrate delays both before first presentation and within healthcare after presentation [16]. Greater recognition of disabling dysmenorrhea, non-menstrual pelvic pain, dyspareunia, bowel or urinary symptoms, and functional impairment is therefore needed before infertility emerges as the principal trigger for assessment [3].

Özcan et al. [22] identified endometriosis-related anxieties and coping strategies among women in Türkiye, including concerns about pregnancy and delayed diagnosis. Our findings are consistent with those concerns, while focusing specifically on women who reported at least two years between symptom onset and diagnosis. Their accounts show how family normalization, clinical dismissal, and patient-led recognition intersected with reproductive uncertainty, treatment decisions, and requests for coordinated care. The syntheses by Davenport et al. [4] and Fryer et al. [6] describe barriers across settings; the present interviews provide situated examples of how these processes connected in one Istanbul hospital sample. These examples do not establish that the experiences are unique to Türkiye or more prevalent than elsewhere.

The second theme illustrates the ambivalent meaning of receiving a diagnosis. Participants experienced relief because the diagnosis named their symptoms, validated their pain, and countered earlier suggestions that they were exaggerating or psychologically unwell. In a New Zealand study, participants with a confirmed diagnosis described relief and validation after their symptoms had previously been dismissed [35]. At the same time, diagnosis led women to reinterpret previous educational, occupational, relational, and reproductive decisions through the lens of lost time. The phrase that diagnosis represented both liberation and lost years captures this dual process. Diagnosis did not mark the end of uncertainty; rather, it shifted uncertainty from the cause of symptoms to questions about recurrence, treatment duration, surgery, ovarian reserve, and future fertility.

Participants described substantial disruption of education, employment, household roles, social participation, and intimate life. These narratives align with recent qualitative evidence describing diagnostic delay, worsening daily life, disrupted intimacy, and uncertainty about having children [10]. They are also supported by studies and reviews demonstrating impaired health-related quality of life and losses through absenteeism and reduced effectiveness while working [7,9]. The present study adds an experiential explanation for these quantitative losses: women did not simply miss work because of pain but also concealed symptoms, anticipated disbelief, cancelled commitments, altered career plans, and attempted to preserve a socially acceptable appearance of functioning.

The third theme demonstrates that fertility-related distress occurred across different reproductive positions. Women actively attempting conception described repeated menstrual disappointment, unsuccessful fertility treatment, treatment burden, and diminishing hope. Younger or unmarried women who were not planning pregnancy described anticipated loss, pressure to make decisions earlier than expected, and fear about future disclosure to a partner. Girard et al. [12] similarly documented how diagnostic delay, distressing care experiences, uncertainty regarding reproductive options, treatment difficulties, and strain within couples shape fertility-related distress. In a small mixed-method study of women with endometriosis and infertility who had experienced unsuccessful assisted reproductive treatment, pain, disruption of daily routines, and dissatisfaction with services were prominent treatment-related dimensions of fertility-related quality of life [14]. These findings suggest that fertility counseling should be based on reproductive intentions and information needs rather than marital status or an assumption that fertility matters only when a woman is currently attempting pregnancy.

Participants also described tension between symptom control and fertility goals. Hormonal suppression could reduce pain but was incompatible with attempts to conceive, while surgery was perceived as both a possible treatment and a potential threat to ovarian reserve. Advice to become pregnant could make reproductive timing feel clinically directed rather than personally chosen. ESHRE guidance emphasizes individualized decision-making that considers symptoms, age, reproductive preferences, previous treatment, ovarian reserve, and the potential benefits and harms of medical, surgical, and fertility interventions [3]. Qualitative research on treatment decisions similarly shows that pain, fertility goals, fear of complications, and the quality of information shape women’s choices [36]. The present findings reinforce the importance of communicating uncertainty honestly without creating unnecessary alarm. Women require clear explanations of what is known, what remains uncertain, and how different options relate to their own priorities.

The fourth theme highlights the central role of communication and trust. Participants contrasted brief, dismissive encounters with consultations in which clinicians listened, investigated, explained the condition in understandable terms, and provided a plan. Mikesell and Bontempo found that patients’ trust was shaped by perceptions of both professional competence and fidelity to the patient’s account; inadequate knowledge was often experienced together with trivialization or dismissal [15]. Recent diagnostic and stigma research likewise identifies symptom normalization, disbelief, and dismissal as barriers to timely and supportive care [16,17]. These accounts suggest that validating communication may help women feel able to disclose concerns and develop trust in clinicians. Whether it changes subsequent help-seeking or care decisions requires further study.

Women’s requests for psychological support and continuity reflect the limitations of fragmented biomedical care. P5’s account illustrated how separate pathways for gynecology, fertility services, pain care, surgery, and mental health support could leave women to coordinate information themselves. Compared with healthy controls, depression and anxiety symptoms are more frequent among women with endometriosis and are associated with pain, fatigue, sleep disturbance, sexual difficulties, and poorer quality of life [11]. Psychological support should not imply that pain is psychogenic; rather, it should form part of integrated care for a chronic condition with emotional, relational, and functional consequences. Guidance, qualitative evidence, and a recent systematic review support multidisciplinary, individualized care that includes reliable information, validation, long-term planning, and social and emotional support [3,19,37]. The requests from P5 and P17 suggest a possible nurse or care-navigator role; its acceptability and effect on care coordination require evaluation.

The final theme shows that coping was both relational and self-directed. Supportive partners and family members helped with household work, treatment procedures, healthcare attendance, and protection from social pressure. However, misunderstanding, fatigue, intrusive questions, and expectations of motherhood could also increase distress. In a qualitative study, male partners described effects of endometriosis on intimacy, childbearing plans, emotional well-being, work, household roles, and household income [38]. Sexual quality of life is also associated with dyspareunia, dysmenorrhea, general health-related quality of life, and employment status [39]. These findings support a couple-sensitive approach when desired by the woman, while avoiding assumptions that partnership or motherhood defines her treatment priorities.

Participants used information-seeking, peer communities, symptom tracking, exercise, dietary modification, meditation, creative activities, psychotherapy, concealment, withdrawal, and complementary or spiritual practices. Márki et al. [19] similarly identified information deficits, uncertainty, complementary treatment use, social support, active coping, and the value of a supportive clinician relationship. Yoon et al. [40] described regaining control, rebuilding daily routines, obtaining emotional support, and taking an active role in treatment as key processes of adaptation. In the present study, however, some coping strategies had mixed consequences. Concealment protected women from immediate judgment but restricted access to support, while complementary practices provided a sense of action yet were sometimes hidden from clinicians because women anticipated disapproval. Nonjudgmental inquiry about all remedies and self-management practices is therefore necessary to identify potential risks and preserve open communication.

Taken together, the findings suggest that improving endometriosis care requires intervention at several levels. The accounts of P18 and P16 suggest that menstrual health education addressing disabling pain and concerns about care-seeking is a plausible approach to test. A cluster-randomized trial found that a brief school-based program improved adolescents’ endometriosis knowledge, confidence, and comfort discussing menstrual health [41]; whether such education shortens diagnostic pathways remains uncertain. Primary and reproductive healthcare professionals require education on the varied presentation of endometriosis, the limitations of negative imaging, and indications for specialist assessment [3,18]. These findings suggest that clinical consultations should assess functional impairment, fertility intentions, sexual and relational experiences, mental health, and treatment priorities, with care tailored to each woman’s goals [36,42]. Written and visual information, continuity of care, timely referral, and coordinated multidisciplinary support may help women understand options and reduce the burden of navigating fragmented services [18,37].

### 4.1. Strengths and Limitations

A strength of this study is its in-depth examination of diagnostic, reproductive, clinical, relational, and coping experiences within the same qualitative inquiry. The resulting sample included women with varied marital, fertility, employment, treatment, and surgical experiences. Independent coding, consensus discussions, repeated analysis, an audit trail of analytic decisions, external expert review, reflexive discussion, and the use of direct quotations strengthened credibility and confirmability.

Several limitations should be considered. Participants were recruited from a single private hospital in Istanbul, and eligibility relied on participants’ self-report of a previous physician diagnosis of endometriosis; medical records, imaging, surgery, and histopathology were not independently reviewed. Chart review was outside this interview-based study, leaving diagnostic status and timing unverified. Reliance on self-report may introduce some uncertainty about diagnostic status and the length of diagnostic delay, which should be considered when interpreting the findings. The sample was restricted to women reporting a diagnostic interval of at least two years. Their experiences may therefore not represent women who remain undiagnosed, receive care in public or rural settings, have shorter or timely diagnostic pathways, or cannot access specialist services. The exclusion of fertility difficulty reported as exclusively attributable to a non-endometriosis cause was also based on participant report, and partner-related diagnoses were not independently verified. Although the eligibility range was 18–40 years, the enrolled sample comprised 18 women aged 22–39 years, and experiences after menopause or in gender-diverse populations were not represented. Interviews conducted in a healthcare setting may have influenced what participants chose to disclose, particularly about dissatisfaction with care, sexuality, family relationships, and complementary practices. Neither transcripts nor the findings were returned to participants for comment or confirmation; consequently, direct participant validation was unavailable. The fourth interview question explicitly mentioned fertility anxiety or uncertainty, although it was conditional on participants having experienced such feelings. This wording may have directed attention to anxiety and influenced how participants framed their accounts; the fertility findings should be read in light of this possibility. Ages at symptom onset, first clinical presentation, and diagnosis were not available for reporting; Table 1 therefore presents age at interview and the self-reported interval from first symptoms to diagnosis. The unavailable participant-level values for time since diagnosis and current treatment or follow-up also limit description of the participants’ current care context. The researchers’ nursing and midwifery backgrounds may have shaped attention to suggestions for a specialist nursing role; this proposal should be treated as a hypothesis for evaluation. The findings are interpretive and do not establish the prevalence of the reported experiences. Nevertheless, the recurrence and coherence of themes across diverse participant profiles provide analytically transferable insights that may inform similar clinical contexts.

### 4.2. Implications for Practice and Research

The findings support a person-centered pathway in which disabling menstrual or pelvic symptoms are investigated without requiring women to repeatedly prove the legitimacy of their pain. A UK survey described the roles and time constraints of endometriosis clinical nurse specialists in specialist centers [43]. The requests of P5 and P17 suggest that a specialist nurse or navigator might help provide information and coordinate care across gynecology, fertility, surgery, pain, and mental health services. A German survey found that most respondents would welcome an endometriosis nurse specialist [44]. The acceptability, feasibility, and effectiveness of this proposed role require evaluation in the present setting.

Future studies should include women from public and private institutions, different regions and socioeconomic groups, adolescents, women without a physician-reported diagnosis, and partners when appropriate. Longitudinal qualitative research could examine how fertility anxiety, treatment preferences, trust, and coping change over time. Intervention studies are also needed to evaluate menstrual health education, clinician training, nurse-led navigation, structured fertility counseling, peer support, and integrated psychological care.

## 5. Conclusions

In this sample of women who reported a diagnostic interval of at least two years, experiences of endometriosis were shaped by the meanings assigned to symptoms within families, communities, and healthcare encounters, as well as by pain and fertility anxiety. Participants described the normalization of severe menstrual pain, restrictions surrounding reproductive healthcare, clinical dismissal or misattribution, and fragmented referral pathways along their routes to diagnosis; many had to persistently advocate for themselves. For these women, diagnosis provided validation but also exposed the emotional and practical costs of lost time. Fertility anxiety ranged from anticipatory uncertainty to repeated treatment-related disappointment and interacted with social expectations, intimate relationships, and treatment choices. These accounts support clearer individualized communication, psychological support, coordinated multidisciplinary care, and accessible nursing guidance for women facing prolonged diagnostic pathways.

## Figures and Tables

**Table 1:** Sociodemographic and Clinical Characteristics of the Participants (N = 18).

ID	Age	Education	Marital	Work	Delay (Years)	Child	Surg.	Try	OR Info	Tx
P1	27	Bachelor’s+	Unmarried	Yes	9	No	Yes	No	Yes	No
P2	33	Bachelor’s+	Married	Yes	7	No	No	Yes	Yes	Yes
P3	24	Associate	Unmarried	Yes	6	No	No	No	No	No
P4	36	Secondary	Married	No	12	Yes	No	Yes	Yes	No
P5	31	Bachelor’s+	Married	Yes	5	No	Yes	Yes	Yes	Yes
P6	29	Secondary	Married	No	8	No	No	Yes	No	No
P7	22	Bachelor’s+	Unmarried	No	4	No	No	No	Yes	No
P8	38	Bachelor’s+	Married	Yes	15	Yes	No	Yes	Yes	Yes
P9	34	Primary	Married	No	10	Yes	No	Yes	No	No
P10	30	Bachelor’s+	Unmarried	Yes	11	No	No	No	Yes	No
P11	35	Associate	Married	Yes	6	No	No	Yes	Yes	Yes
P12	26	Bachelor’s+	Married	Yes	3	No	No	No	Yes	No
P13	32	Secondary	Married	No	9	No	No	Yes	No	Yes
P14	28	Bachelor’s+	Unmarried	Yes	2	No	No	No	Yes	No
P15	39	Bachelor’s+	Married	Yes	14	Yes	No	Yes	Yes	Yes
P16	25	Secondary	Married	No	7	No	No	Yes	No	No
P17	30	Bachelor’s+	Married	Yes	8	No	No	Yes	Yes	No
P18	27	Secondary	Married	No	6	No	No	Yes	No	No

Note: ID, participant identifier; Bachelor’s+, bachelor’s degree or higher; Delay, interval from symptom onset to the diagnosis reported by the participant; Work, currently employed; Try, previous pregnancy attempt; OR info, information received about ovarian reserve; Tx, fertility treatment; Surg., surgery for endometriosis. Participants are identified by codes P1–P18. The descriptive form also asked about time since diagnosis and current treatment or follow-up; participant-level responses were unavailable for reporting.

**Table 2:** Themes and Subthemes Derived from the Interviews with Women Diagnosed with Endometriosis.

Theme	Subthemes
Experiences of diagnostic delay	Normalization and delayed help-seeking Clinical dismissal and symptom misattribution Patient-led recognition and persistence Fertility difficulty as a route to diagnosis
Meaning and consequences of the delayed diagnosis	Relief and validation after diagnosis Anger, regret, and lost time Disruption of education, work, family, and intimate life Ongoing uncertainty and mistrust
Fertility anxiety and reproductive decision-making	Anticipatory fear and perceived reproductive time pressure Repeated disappointment during attempts to conceive Motherhood expectations and feelings of inadequacy Tension between symptom treatment and fertility goals
Experiences and expectations regarding healthcare	Uneven information about endometriosis and ovarian reserve Importance of clear and individualized communication Need for psychological support Need for coordinated multidisciplinary care and nursing support
Social support and coping strategies	Support and strain within partner and family relationships Information-seeking, peer support, and self-management Concealment, withdrawal, and protection from social pressure Use of complementary and spiritual practices

## Data Availability

The interview transcripts are not publicly available because they contain sensitive personal narratives and contextual information that could compromise participant confidentiality. Anonymized quotations supporting the findings are provided in the manuscript.

## Supplementary Materials

The following supporting information can be downloaded at: https://www.mdpi.com/article/10.3390/jcm15218510/s1, Table S1: COREQ 32-Item Checklist; Table S2: Descriptive Information Form and Semi-Structured Interview Guide; Table S3: Coding Tree for the Thematic Analysis.

## Abbreviations

The following abbreviations are used in this manuscript:

COREQ	Consolidated Criteria for Reporting Qualitative Research

## References

[1] Zondervan K.T., Becker C.M., Koga K., Missmer S.A., Taylor R.N., Viganò P. (2018). Endometriosis. Nat. Rev. Dis. Primers.

[2] Zondervan K.T., Becker C.M., Missmer S.A. (2020). Endometriosis. N. Engl. J. Med..

[3] Becker C.M., Bokor A., Heikinheimo O., Horne A., Jansen F., Kiesel L., King K., Kvaskoff M., Nap A., Petersen K. (2022). ESHRE Endometriosis Guideline Group. ESHRE guideline: Endometriosis. Hum. Reprod. Open.

[4] Davenport S., Smith D., Green D.J. (2023). Barriers to a timely diagnosis of endometriosis: A qualitative systematic review. Obstet. Gynecol..

[5] De Corte P., Klinghardt M., von Stockum S., Heinemann K. (2025). Time to diagnose endometriosis: Current status, challenges and regional characteristics—A systematic literature review. BJOG.

[6] Fryer J., Mason-Jones A.J., Woodward A. (2025). Understanding diagnostic delay for endometriosis: A scoping review using the social-ecological framework. Health Care Women Int..

[7] Andysz A., Jacukowicz A., Merecz-Kot D., Najder A. (2018). Endometriosis—The challenge for occupational life of diagnosed women: A review of quantitative studies. Med. Pr..

[8] Moradi M., Parker M., Sneddon A., Lopez V., Ellwood D. (2014). Impact of endometriosis on women’s lives: A qualitative study. BMC Women’s Health.

[9] Mousa M., Al-Jefout M., Alsafar H., Becker C.M., Zondervan K.T., Rahmioglu N. (2021). Impact of endometriosis in women of Arab ancestry on: Health-related quality of life, work productivity, and diagnostic delay. Front. Glob. Women’s Health.

[10] Rea T., Giampaolino P., Simeone S., Pucciarelli G., Alvaro R., Guillari A. (2020). Living with endometriosis: A phenomenological study. Int. J. Qual. Stud. Health Well-Being.

[11] van Barneveld E., Manders J., van Osch F.H.M., van Poll M., Visser L., van Hanegem N., Lim A.C., Bongers M.Y., Leue C. (2022). Depression, anxiety, and correlating factors in endometriosis: A systematic review and meta-analysis. J. Women’s Health.

[12] Girard E., Mazloum A., Navarria-Forney I., Pluchino N., Streuli I., Cedraschi C. (2023). Women’s lived experience of endometriosis-related fertility issues. PLoS ONE.

[13] Heng F.W., Shorey S. (2022). Experiences of endometriosis-associated infertility among women and their partners: A qualitative systematic review. J. Clin. Nurs..

[14] Zarbo C., Compare A., Frigerio L., Secomandi R., Bellia A., Betto E., Candeloro I., Dessi V., Malandrino C., Peduzzi C. (2023). Going beyond the surface: A mixed-method exploration of infertility-related quality of life of women with endometriosis. Psychol. Health Med..

[15] Mikesell L., Bontempo A.C. (2023). Healthcare providers’ impact on the care experiences of patients with endometriosis: The value of trust. Health Commun..

[16] Requadt E., Nahlik A.J., Jacobsen A., Ross W.T. (2024). Patient experiences of endometriosis diagnosis: A mixed methods approach. BJOG.

[17] Sims O.T., Gupta J., Missmer S.A., Aninye I.O. (2021). Stigma and endometriosis: A brief overview and recommendations to improve psychosocial well-being and diagnostic delay. Int. J. Environ. Res. Public Health.

[18] van der Zanden M., de Kok L., Nelen W.L.D.M., Braat D.D.M., Nap A.W. (2021). Strengths and weaknesses in the diagnostic process of endometriosis from the patients’ perspective: A focus group study. Diagnosis.

[19] Márki G., Vásárhelyi D., Rigó A., Kaló Z., Ács N., Bokor A. (2022). Challenges of and possible solutions for living with endometriosis: A qualitative study. BMC Women’s Health.

[20] Darici E., Kemahlı M.N.C., Bahat P.Y., Yücel B., Oral E. (2023). Validation of the Turkish version of Endometriosis Health Profile questionnaire (EHP-30) to evaluate the quality of life in women with endometriosis. Facts Views Vis. ObGyn.

[21] Dişsiz M., İşbay B., Aydın S., Bingöl D., Öner Ö., Karakuş R. (2024). The impact of endometriosis on women’s quality of life and sexual life. Arch. Health Sci. Res..

[22] Özcan H., Bilgiç D., Köksaldi E. (2025). Women affected by endometriosis: Their anxieties and coping methods. J. Eval. Clin. Pract..

[23] Sandelowski M. (2000). Whatever happened to qualitative description?. Res. Nurs. Health.

[24] Braun V., Clarke V. (2021). Can I use TA? Should I use TA? Should I not use TA? Comparing reflexive thematic analysis and other pattern-based qualitative analytic approaches. Couns. Psychother. Res..

[25] Tong A., Sainsbury P., Craig J. (2007). Consolidated criteria for reporting qualitative research (COREQ): A 32-item checklist for interviews and focus groups. Int. J. Qual. Health Care.

[26] Palinkas L.A., Horwitz S.M., Green C.A., Wisdom J.P., Duan N., Hoagwood K. (2015). Purposeful sampling for qualitative data collection and analysis in mixed method implementation research. Adm. Policy Ment. Health.

[27] Saunders B., Sim J., Kingstone T., Baker S., Waterfield J., Bartlam B., Burroughs H., Jinks C. (2018). Saturation in qualitative research: Exploring its conceptualization and operationalization. Qual. Quant..

[28] Agarwal S.K., Chapron C., Giudice L.C., Laufer M.R., Leyland N., Missmer S.A., Singh S.S., Taylor H.S. (2019). Clinical diagnosis of endometriosis: A call to action. Am. J. Obstet. Gynecol..

[29] Facchin F., Saita E., Barbara G., Dridi D., Vercellini P. (2018). “Free butterflies will come out of these deep wounds”: A grounded theory of how endometriosis affects women’s psychological health. J. Health Psychol..

[30] Braun V., Clarke V. (2006). Using thematic analysis in psychology. Qual. Res. Psychol..

[31] Nowell L.S., Norris J.M., White D.E., Moules N.J. (2017). Thematic analysis: Striving to meet the trustworthiness criteria. Int. J. Qual. Methods.

[32] Guba E.G. (1981). Criteria for assessing the trustworthiness of naturalistic inquiries. ECTJ.

[33] Korstjens I., Moser A. (2018). Series: Practical guidance to qualitative research. Part 4: Trustworthiness and publishing. Eur. J. Gen. Pract..

[34] Berger R. (2015). Now I see it, now I don’t: Researcher’s position and reflexivity in qualitative research. Qual. Res..

[35] Ellis K., Munro D., Wood R. (2022). The experiences of endometriosis patients with diagnosis and treatment in New Zealand. Front. Glob. Women’s Health.

[36] Metzemaekers J., Slotboom S., Sampat J., Vermolen P., Smeets M.J.G.H., van den Akker-van Marle M.E., Maas J., Bakker E.C., Nijkamp M., Both S. (2021). Crossroad decisions in deep endometriosis treatment options: A qualitative study among patients. Fertil. Steril..

[37] Fang Q.Y., Campbell N., Mooney S.S., Holdsworth-Carson S.J., Tyson K. (2024). Evidence for the role of multidisciplinary team care in people with pelvic pain and endometriosis: A systematic review. Aust. N. Z. J. Obstet. Gynaecol..

[38] Culley L., Law C., Hudson N., Mitchell H., Denny E., Raine-Fenning N. (2017). A qualitative study of the impact of endometriosis on male partners. Hum. Reprod..

[39] van Poll M., van Barneveld E., Aerts L., Maas J.W.M., Lim A.C., de Greef B.T.A., Bongers M.Y., van Hanegem N. (2020). Endometriosis and sexual quality of life. Sex. Med..

[40] Yoon Y., Park M.A., Park S. (2021). Seeking adaptation from uncertainty: Coping strategies of South Korean women with endometriosis. Res. Nurs. Health.

[41] Wahl K., Albert A., Larente M., Lopez de Arbina E., Kennedy L., Sutherland J.L., Imtiaz S., Orr N.L., Allaire C., Yong P.J. (2024). Does menstrual health and endometriosis education affect knowledge among middle and secondary school students? A cluster-randomised controlled trial. J. Obstet. Gynaecol. Can..

[42] Omtvedt M., Bean E., Hald K., Raasholm Larby E., Majak G.B., Tellum T. (2022). Patients’ and relatives’ perspectives on best possible care in the context of developing a multidisciplinary center for endometriosis and adenomyosis: Findings from a national survey. BMC Women’s Health.

[43] Norton W., Mitchell H., Holloway D., Law C. (2020). The role of Endometriosis Clinical Nurse Specialists in British Society for Gynaecological Endoscopy registered centres: A UK survey of practice. Nurs. Open.

[44] Lukac S., Kässens V.M., Barzel A., Kloss T., Mayer I., Janni W., Hancke K., Dayan D. (2025). Evaluation of the need for the integration of a nurse specialist (EndoNurse) into the interdisciplinary care of patients with endometriosis: Cross-sectional study. Int. J. Nurs. Stud. Adv..

