# Supplementary material for: Experiences of Diagnostic Delay and Fertility Anxiety Among Women with Endometriosis: A Qualitative Study
Source: J Clin Med. 2026 Nov 2;15(21):8510. doi: 10.3390/jcm15218510 (PMC13764372; doi:10.3390/jcm15218510)
Supplement: Supplementary file 1 [file jcm-15-08510-s001.zip › Supplementary Table S1 .pdf]

Table S1: COREQ 32-Item Checklist

Consolidated Criteria for Reporting Qualitative Research

Manuscript: Experiences of Diagnostic Delay and Fertility Anxiety among Women with Endometriosis: A Qualitative Study

Reference: 25. Tong, A.; Sainsbury, P.; Craig, J. Consolidated criteria for reporting qualitative research (COREQ): a 32-item checklist for interviews and focus groups. Int. J. Qual. Health Care 2007, 19(6), 349–357. https://doi.org/10.1093/intqhc/mzm042

No.	Item	Guide question or description	Reported in the manuscript
	**Domain 1: Research team and reflexivity**		
1	Interviewer/facilitator	Which author(s) conducted the interviews?	Materials and Methods—Data Collection (S.A.D. and R.O.Y.)
2	Credentials	What were the researchers' credentials?	Materials and Methods—Researcher Characteristics and Reflexivity (S.A.D., MSc; R.O.Y., PhD).
3	Occupation	What was their occupation at the time of the study?	Materials and Methods—Data Collection; Researcher Characteristics and Reflexivity (nurses by professional training; academic appointments in departments of midwifery).
4	Gender	Were the researchers male or female?	Materials and Methods—Researcher Characteristics and Reflexivity (both female).
5	Experience and training	What experience or training did the researchers have?	Materials and Methods—Data Collection; Researcher Characteristics and Reflexivity (clinical and qualitative research experience in women's health).
6	Relationship established	Was a relationship established before study commencement?	Materials and Methods—Participants and Setting; Data Collection (no prior relationship).
7	Participant knowledge of interviewer	What did participants know about the researchers?	Materials and Methods—Data Collection (academic researchers independent of participants' clinical care; participation would not affect care).
8	Interviewer characteristics	What characteristics were reported about the interviewers?	Materials and Methods—Researcher Characteristics and Reflexivity.
	**Domain 2: Study design**		
9	Methodological orientation and theory	What methodological orientation was stated?	Materials and Methods—Study Design and Data Analysis (qualitative descriptive design; codebook approach to thematic analysis).
10	Sampling	How were participants selected?	Materials and Methods—Participants and Setting (purposive, criterion-based sampling).
11	Method of approach	How were participants approached?	Materials and Methods—Participants and Setting (clinic attendees were asked about prior diagnosis and interest; researchers assessed eligibility).
12	Sample size	How many participants were included?	Materials and Methods—Participants and Setting; Results (N = 18).
13	Non-participation	How many declined or withdrew?	The numbers of women approached, those who declined before consent, and those excluded during eligibility screening were not recorded. Eighteen eligible volunteers were enrolled; no enrolled participant withdrew.
14	Setting of data collection	Where were data collected?	Materials and Methods—Data Collection (private room in the gynecology outpatient clinic).
15	Presence of non-participants	Was anyone else present?	Materials and Methods—Data Collection (no one other than the participant and interviewer).
16	Description of sample	What are the important sample characteristics?	Results—Table 1.
17	Interview guide	Were questions, prompts, and guides provided?	Materials and Methods—Data Collection; Supplementary Table S2.
18	Repeat interviews	Were repeat interviews conducted?	Materials and Methods—Data Collection (one interview per participant; no repeat interviews).

No.	Item	Guide question or description	Reported in the manuscript
19	Audio/visual recording	Was audio or visual recording used?	Materials and Methods—Data Collection (audio-recorded with permission).
20	Field notes	Were field notes made during or after interviews?	Materials and Methods—Data Collection (brief field notes were made during and immediately after interviews and considered with transcripts).
21	Duration	What was the interview duration?	Materials and Methods—Data Collection (approximately 50–60 min).
22	Data saturation	Was saturation discussed?	Materials and Methods—Participants and Setting (interview-by-interview comparisons; final interviews added no substantive changes to the codebook or developing themes; recruitment ended after 18 interviews).
23	Transcripts returned	Were transcripts returned for comment or correction?	Materials and Methods—Data Collection (transcripts were not returned for comment or correction).
	**Domain 3: Analysis and findings**		
24	Number of data coders	How many coders coded the data?	Materials and Methods—Data Analysis (two researchers).
25	Description of coding tree	Was a coding tree described?	Materials and Methods—Data Analysis; Results—Table 2; Supplementary Table S3.
26	Derivation of themes	Were themes predetermined or data-derived?	Materials and Methods—Data Analysis (interview guide provided broad topics; participants' accounts informed the codes and subthemes, which were organized into five themes).
27	Software	What software was used to manage data?	Materials and Methods—Data Analysis (manual coding; no qualitative data-analysis software).
28	Participant checking	Did participants provide feedback on findings?	Materials and Methods—Trustworthiness; Strengths and Limitations (not undertaken).
29	Quotations presented	Were participant quotations used to illustrate themes?	Results—Sections 3.1–3.5.
30	Data and findings consistent	Was there consistency between data and findings?	Results—Sections 3.1–3.5; quotations are linked to each subtheme.
31	Clarity of major themes	Were major themes clearly presented?	Results—Table 2 and Sections 3.1–3.5 (five themes).
32	Clarity of minor themes	Were diverse cases or minor themes described?	Results—Table 2 and Sections 3.1–3.5 (subthemes and contrasting accounts).
