# Supplementary material for: Experiences of Diagnostic Delay and Fertility Anxiety Among Women with Endometriosis: A Qualitative Study
Source: J Clin Med. 2026 Nov 2;15(21):8510. doi: 10.3390/jcm15218510 (PMC13764372; doi:10.3390/jcm15218510)
Supplement: Supplementary file 1 [file jcm-15-08510-s001.zip › Supplementary Table S2.pdf]

Table S2: Descriptive Information Form and Semi-Structured Interview Guide

The interviews were conducted in Turkish and lasted approximately 50–60 min. The English wording below is a faithful translation of the original 12-item descriptive form and five main open-ended questions used in the study. Follow-up prompts were used when clarification or elaboration was needed.

Part A Descriptive Information Form

No.	Item
1	Age
2	Educational level: Primary education; Secondary education; Associate degree; Bachelor's degree or higher
3	Marital status: Married; Unmarried
4	Employment status: Employed; Not employed
5	How many years elapsed between the onset of your first pain or symptoms and the diagnosis you reported?
6	Do you currently have any children? Yes; No
7	Have you undergone surgery because of endometriosis or an endometrioma? Yes; No
8	Have you ever attempted to become pregnant? Yes; No
9	Have you been informed that your condition may reduce ovarian reserve? Yes; No
10	Have you received any fertility treatment? Yes; No
11	How many years have passed since your endometriosis diagnosis?
12	Are you currently receiving treatment or follow-up for endometriosis? If yes, please specify.

Part B Semi Structured Interview Guide

No.	Main interview question
1	Could you describe the period from the onset of your first symptoms until you received the diagnosis you reported?
2	What did you experience during this period?
3	How, if at all, has having endometriosis affected your thoughts, feelings, or plans regarding future fertility or having children?
4	If you have experienced anxiety or uncertainty about fertility, how have you dealt with these feelings, if at all?
5	What kind of approach or support would you like to receive from healthcare professionals or healthcare services during this process?

Closing invitation (not counted among the five main questions): Is there anything else you would like to add about your experience with endometriosis, your path to diagnosis, or your fertility anxiety?
