# Supplementary material for: Experiences of Diagnostic Delay and Fertility Anxiety Among Women with Endometriosis: A Qualitative Study
Source: J Clin Med. 2026 Nov 2;15(21):8510. doi: 10.3390/jcm15218510 (PMC13764372; doi:10.3390/jcm15218510)
Supplement: Supplementary file 1 [file jcm-15-08510-s001.zip › Supplementary Table S3.pdf]

Supplementary Table S3

Coding Tree for the Thematic Analysis

The coding tree shows how data-near initial codes were grouped into intermediate categories, refined into subthemes, and synthesized into the five final themes. Coding was conducted manually and documented in the evolving codebook and analytic notes. English formulations preserve the meanings expressed in the Turkish interviews. For example, the initial codes “severe menstrual pain treated as ordinary” and “family history used to normalize symptoms” were grouped under the intermediate category “familial normalization”, which contributed to the subtheme “normalization and delayed help-seeking” within the final theme “experiences of diagnostic delay”.

Final theme	Subtheme	Intermediate categories	Initial codes
Experiences of diagnostic delay	Normalization and delayed help-seeking	Familial normalization; gendered endurance; barriers related to unmarried status	Severe menstrual pain treated as ordinary; expectation that pain would resolve after marriage or childbirth; family history used to normalize symptoms; delayed first consultation; fear that examination could affect virginity
Experiences of diagnostic delay	Clinical dismissal and symptom misattribution	Episodic symptom control; misattribution across specialties	Analgesics without investigation; normal test reassurance; infection treatment; bowel diagnosis; psychological attribution; vaginismus label; postoperative-adhesion explanation; repeated emergency visits
Experiences of diagnostic delay	Patient-led recognition and persistence	Self-education; persistence across consultations	Learning about endometriosis from peers or online sources; asking clinicians about endometriosis; changing physicians; feeling responsible for one’s own diagnosis; being dismissed for internet searching
Experiences of diagnostic delay	Fertility difficulty as a route to diagnosis	Reproductive investigation making disease visible	Diagnosis during infertility assessment; imaging at assisted reproduction centers; pain overlooked until non-conception; questioning earlier insemination without diagnosis
Meaning and consequences of the delayed diagnosis	Relief and validation after diagnosis	Personal validation; relational legitimacy	Realizing the pain was real; being able to name the condition; explaining incapacity to others; countering labels of exaggeration, anxiety, weakness, or laziness; family recognition after diagnosis
Meaning and consequences of the delayed diagnosis	Anger, regret, and lost time	Retrospective reinterpretation; lost-time narrative	Relief followed by anger; mourning lost years; repeated ‘if only’ thinking; regret about missed examinations or ineffective treatment; self-blame; anger about delayed recognition
Meaning and consequences of the delayed diagnosis	Disruption of education, work, family, and intimate life	Functional loss; concealment; role disruption	School and examination absence; delayed education; reduced productivity; lost promotion; leaving employment; financial strain; impaired housework and parenting; sexual avoidance; cancelled social plans
Meaning and consequences of the delayed diagnosis	Ongoing uncertainty and mistrust	Chronic vigilance; treatment uncertainty; weakened trust	Uncertainty about medication duration and side effects; fear of recurrence or progression; uncertainty about surgery; inconsistent clinical opinions; mistrust after missed diagnoses; anxiety before follow-up imaging
Fertility anxiety and reproductive decision-making	Anticipatory fear and perceived reproductive time pressure	Anticipated infertility; premature decision pressure	Fear about future conception; anxiety about ovarian reserve; considering fertility preservation; pressure to marry or conceive; future disclosure to a partner; receiving advice not to wait
Fertility anxiety and reproductive decision-making	Repeated disappointment during attempts to conceive	Hope and grief across repeated attempts	Anxiety during pregnancy testing; menstruation as recurring loss; failed insemination or IVF; treatment burden; diminishing hope; linking reduced chances to delayed diagnosis
Fertility anxiety and reproductive decision-making	Motherhood expectations and feelings of inadequacy	Gendered identity; family-completeness expectations	Feeling incomplete or deficient; pressure for a first or second child; guilt about not providing a sibling; comparison with pregnant peers; intrusive questions from relatives
Fertility anxiety and reproductive decision-making	Tension between symptom treatment and fertility goals	Competing clinical priorities; constrained reproductive autonomy	Unable to use hormonal suppression while trying to conceive; weighing surgery against ovarian reserve; pregnancy framed as treatment; disease influencing timing of parenthood

Final theme	Subtheme	Intermediate categories	Initial codes
Experiences and expectations regarding healthcare	Uneven information about endometriosis and ovarian reserve	Information gaps; delayed counselling; self-directed searching	No clear explanation of disease course or treatment duration; no written resources; learning reserve status late; not knowing what to ask; frightening online statistics; fertility-risk information without follow-up
Experiences and expectations regarding healthcare	Importance of clear and individualized communication	Listening and validation; accessible explanation; anticipatory guidance	Detailed symptom history; clinician-initiated fertility discussion; diagrams; written medication instructions; age- and intention-sensitive information; clear risks and next steps
Experiences and expectations regarding healthcare	Need for psychological support	Emotional needs left outside routine care; biomedical focus	No enquiry about emotional well-being; crying after failed treatment; depression after prolonged pain; couple strain; seeking therapy independently; desire for nursing or psychological support
Experiences and expectations regarding healthcare	Need for coordinated multidisciplinary care and nursing support	Fragmented services; navigation and continuity needs	Separate pain, fertility, surgery, and mental health pathways; repeated referrals; contradictory opinions; lack of follow-up; desire for an accessible endometriosis nurse; coordinated referrals and staged plans
Social support and coping strategies	Support and strain within partner and family relationships	Practical and emotional support; reproductive pressure; relational fatigue	Partner attending appointments; administering injections; household help; defending against relatives; improved understanding after clinical explanation; partner fatigue; family and in-law pressure
Social support and coping strategies	Information-seeking, peer support, and self-management	Restoring control; belonging; structured self-management	Reading articles; online support groups; symptom tracking; exercise; dietary modification; meditation; art; psychotherapy; feeling less alone through peer connection
Social support and coping strategies	Concealment, withdrawal, and protection from social pressure	Protection from judgment; resulting isolation	Hiding fertility treatment at work; withholding fertility information from family; no longer discussing pain; avoiding social events; withdrawing from friends with children; reduced access to support
Social support and coping strategies	Use of complementary and spiritual practices	Family and peer advice; unmonitored use; uncertain safety	Herbal teas; onion remedies; chasteberry; royal jelly and pollen; dietary restrictions; prayer; religiously meaningful products; non-disclosure to clinicians; use despite uncertainty
